# Additional findings of tibial dysplasia in a male with orofaciodigital syndrome type XVI

**DOI:** 10.1038/s41439-022-00187-9

**Published:** 2022-03-31

**Authors:** Yasutsugu Chinen, Sadao Nakamura, Kumiko Yanagi, Takuya Kaneshi, Hideki Goya, Tomohide Yoshida, Kazuhito Satou, Tadashi Kaname, Kenji Naritomi, Koichi Nakanishi

**Affiliations:** 1 Department of Child Health and Welfare (Pediatrics), Graduate School of Medicine, University of the Ryukyus, Nishihara, Okinawa Japan; 2 Genetic Counseling Unit, University of the Ryukyus Hospital, Nishihara, Okinawa Japan; 3 Department of Genome Medicine, National Center for Child Health and Development, Tokyo, Japan; 4 Okinawa Nanbu Habilitation and Medical Center, Naha, Okinawa Japan

**Keywords:** Genetic counselling, Disease genetics

## Abstract

We describe the case of a male patient with orofaciodigital (OFD) syndrome type XVI with a homozygous variant of *TMEM107* (p.Phe106del) and the additional findings of tibial dysplasia, which is a pivotal finding of OFD syndrome type IV. His family history included two fetuses with anencephaly with or without cleft lip/palate and polydactyly with no genetic information. Careful attention should be given to the interpretation of this rare pattern.

Orofaciodigital (OFD) syndromes are rare heterogenetic disorders that are characterized by malformations of the face, oral cavity, digits, and other body parts, and they are also disorders of the central nervous system, eyes, and kidneys. OFD syndromes are clinically classified into 15 Online Mendelian Inheritance in Man (OMIM)-established subtypes, such as OFD syndrome type I (polycystic kidney), OFD syndrome type IX (retinal abnormalities), OFD syndrome type IV (tibial dysplasia), and OFD syndrome type VI (mesoaxial polydactyly, vermis hypoplasia, and molar tooth sign [MTS])^1^. OFD syndrome type XVI (MIM #617563) was first described by Shylo et al. (2016); it is caused by homozygous or compound heterozygous mutations in the *TMEM107* gene on chromosome 17p13. The clinical features of OFD syndrome type XVI mainly include postaxial polydactyly of the hands and feet, vermis hypoplasia, MTS, retinopathy, apnea/hyperpnea, and developmental delay^2–5^. OFD syndromes are classified under ciliopathies, which also include Joubert syndrome (JBS), Meckel–Gruber syndrome (MKS), Bardet–Biedl syndrome, nephronophthisis (NPHP), and several chondrodysplasias^6^. Ciliopathies caused by *TMEM107* gene dysfunction are Meckel syndrome-13 (MKS13) and Joubert syndrome-29 (JBTS29) (MIM #617562)^3,5^. TMEM107 encodes a protein that is localized to the transition zone (TZ) in the proximal region of the ciliary axoneme. The TZ facilitates a protein diffusion barrier at the ciliary base, thereby regulating ciliary composition and signaling^4^.

The patient was a male who was born as the fifth pregnancy, second child, to a healthy, nonconsanguineous couple in Okinawa, Japan. The father and mother were aged 28 years at the time of his birth. His siblings included an elder healthy boy. The mother had had three pregnancy terminations, including two fetuses with anencephaly (Fig. 1a: III-2, III-4) and one with cleft lip/palate and polydactyly (Fig. 1a: III-4). At 28 weeks and 6 days of gestation, the patient’s femur length was 42 mm (−2.9 SD), and his humeral length was 38 mm. These measurements are equivalent to those typically seen at 24 gestational weeks. At 32 gestational weeks, fetal magnetic resonance imaging (MRI) detected polydactyly and limb shortening. At 41 gestational weeks, his delivery was uneventful. He weighed 3074 g (−0.6 SD) and measured 48.5 cm (−1.0 SD) in length. His occipitofrontal circumference (OFC) was 35.0 cm (0.8 SD). Echocardiography and abdominal echoes revealed no abnormalities. At 5 months old, he was referred to our clinic for failure to thrive and developmental delay. He had a broad forehead, hypertelorism, short nose, anteverted nares, broad nasal bridge, broad nasal tip, thin upper lip, hyperplastic oral frenula, high-arched palate, lobulated tongue, oral lingual nodules, tongue hamartomas, puffy cheeks, micrognathia, bilateral postaxial polydactyly of the hands, bilateral mesoaxial polydactyly of the feet, short limbs, left inguinal hernia, buried penis, and migrating testis (Fig. 1a–h). Occasionally, sudden temporary hyperpnea occurred. He also had strabismus. Radiographic imaging at 48 days showed the 6^th^ finger, including two proximal phalanxes and one distal phalanx (Fig. 1i), Y-shaped 5^th^ metacarpal (Fig. 1i) and broad 5^th^ metacarpal (Fig. 1j), polydactyly of feet without fused bone (Fig. 1k, l), shortly bowed tibia and mildly flared femoral metaphysis (Fig. 1m, n). These findings showed improvement at 14 months (Fig. 1o). MRI showed cerebellar vermis hypoplasia and a molar tooth sign (Fig. 1p, q). Additionally, an echocardiogram, abdominal echogram, and auditory brainstem response test revealed no apparent abnormalities. At 2 years old, his weight was 8219 g (−2.9 SD), length was 70.8 cm (−4.9 SD), and the OFC was 48.5 cm (0.1 SD). Eventually, he underwent bilateral orchidopexy, Potts’ operation for left inguinal herniation, and resection of two tongue hamartomas and ectopic upper/lower labial zonules. His ability to eat was improved after the surgery.

**Fig. 1:**
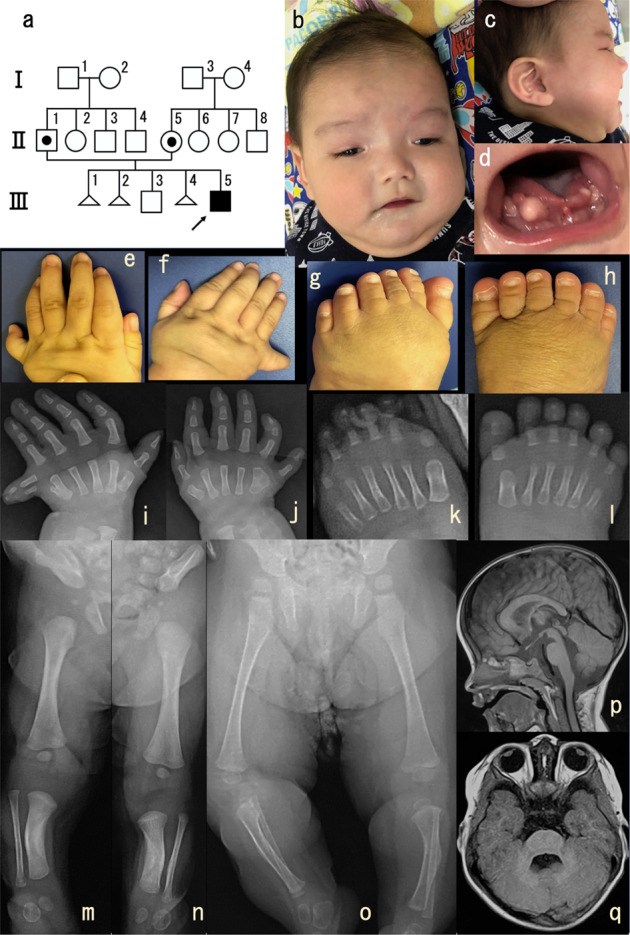
Patient summary and imaging study. **a** Pedigree: III-1, spontaneous termination of pregnancy; III-2, anencephaly, induced termination of pregnancy; III-4, anencephaly, cleft lip/palate, polydactyly, and induced termination of pregnancy. **b**, **c** Patient’s appearance at 6 months old. **d** Patient’s lobulated tongue, oral lingual nodules, and oral hamartomas at 6 months old. **e**, **f** Patient’s bilateral postaxial polydactyly of the hands at 6 months old. **g**, **h** Patient’s bilateral mesoaxial polydactyly of the feet at 6 months old. **i**–**l** Patient’s bilateral polydactyly of the hands **i**, **j** and feet **k**, **l** at 48 days imaged via radiography. **m**–**o** Patient’s lower limbs imaged via radiography [**m**, **n**: at 48 days, **o** at 14 months]. **p**, **q** Patient’s magnetic resonance imaging of the brain at 15 months showing cerebellar vermis hypoplasia and molar tooth sign.

After his parents provided written informed consent, we conducted trio-based whole-exome sequencing using the Sure Select Human All Exon V6 kit (Agilent Technologies, Santa Clara, CA) and HiSeq2500 (Illumina, San Diego, CA) and in silico analyses, as described previously^7^. After performing filtering in the in silico analyses, we identified a homozygous deletion in exon 4 of *TMEM107*, NM_032354: c.316_318del (p.Phe106del). This mutation had been previously described and was indicated to cause disruptions in cilia formation and length^3,4^. Located in the third transmembrane domain of *TMEM107*, Phe106 is highly conserved from fungi to humans, emphasizing that the residue is probably functionally important. The patient’s parents had a heterozygous variant of p.Arg275Gln within *TMEM107*. However, this variant was not found in the Japanese Genome Database of Human Genetic Variation (http://www.hgvd.genome.med.kyoto-u.ac.jp) or the Single Nucleotide Polymorphism Database (http://www.ncbi.nlm.gov/SNP). Instead, it was found in the Genome Aggregation Database (https://gnomad.broadinstitute.org). Its frequency is extremely low (0.00002122) [allele count: 6/282768, east Asian: 0/19952, European (non-Finnish): 6/129126)], with zero homozygotes reported. The ACMG-AMP guidelines have classified this variant as pathogenic (PVS1, PS3, PM3, PM4, PP3, and PP4). This study was performed in accordance with the standards of the Ethics Committee of the Ryukyus Graduate School of Medicine (Okinawa, Japan).

The patient’s final diagnosis was OFD syndrome type XVI based on clinical and molecular findings, as he did not have the metacarpal abnormalities with central polydactyly that are seen in OFD syndrome type VI. The clinical characteristics of the previously reported cases of *TMEM107* gene variants are summarized in Table 1^3–6,8,9^. Although case 1 had insufficient clinical findings of MKS, all five cases, including the siblings, exhibited various phenotypes involving the oral and central nervous systems. Both cases 4 and 5 had the same gene variants but different findings. In case 5, we observed tibial dysplasia and mildly flared femoral metaphysis, which were not previously reported in OFD syndrome type XVI. Tibial dysplasia is a pivotal feature of OFD syndrome type IV^10,11^. In Table 1, five out of six patients had molar tooth signs, which are important clinical data for OFD syndrome type VI^12^.

**Table 1:** Clinical characteristics of *TMEM107* gene variations in reported cases.

	Shaheen et al. (2015)	Bruel et al. (2017)/Lambacher et al. (2016)/Darmency-Stamboul et al. (2013)		Lambacher et al. (2016)	Iglesias et al. (2014)/Shylo et al. (2016)	Present case
Cases	1	2a	2b	3	4	5
Clinical subtype	MKS	OFDVI	OFDVI	JBS	atypical OFD	OFDXVI
Gene analysis results	p.Ser92Cysfs*7/p.Ser92Cysfs*7	p.Glu45Gly/ p.Glu45Gly	p.Glu45Gly/ p.Glu45Gly	p.Leu134Phefs*8/ p.Phe106del	p.Phe106del/ p.Phe106del	p.Phe106del/ p.Phe106del
Sex	Male	Female	Female	Male	NA	Male
Age at last follow-up	Still-born	9 years	9 years	22 years	2 years	3 years
Origin	Saudi Arabia	Turkey	Turkey	Caribbean	NA	Japan
Consanguinity	+	+	+	−	NA	−
Cleft lip	−	−	−	−	−	−
Cleft palate	NA	−	−	−	−	+
Lobulated tongue	NA	−	−	−	−	+
Abnormal frenula	NA	+	+	−	NA	+
Lingual harmatomas	NA	+	+	−	+	+
Micro/retroagnathia	+	−	−	−	+	+
Hypertelorism	+	+	+	−	NA	+
Flat nasal bridge	+	NA	NA	NA	+	+
Retinopathy	NA	+	+	+	NA	−
Low-set ears	+	+	+	−	+	+
Hand/polydactyly	+	+	+	−	+	+
Foot/polydactyly	+	+	+	−	+	+
Apnea/hyperpnea	NA	+	+	−	NA	+
Ataxia	NA	+	+	+	NA	+
Oculomotor apraxia	NA	+	+	+	NA	−
Developmental delay	NA	+	+	+	+	+
Cerebellar hypoplasia	NA	+	+	+	−	+
Molar tooth sign	NA	+	+	+	−	+
Heterotopia	NA	+	+	−	−	−
Polymicrogyria	NA	−	+	−	−	−
Tibial dysplasia	NA	−	−	−	−	+

*NA* not available, *MKS* Meckel–Gruber syndrome, *JBS* Joubert syndrome, *OFD* Orofaciodigital syndrome.

Bruel et al. (2017) suggested a novel classification of OFD syndrome in which the molar tooth sign is an associated clinical feature of OFD syndrome type VI that is caused by genetic variants in *TMEM107*, *TMEM216*, *TMEM231*, *TMEM138*, *C5orf42*, and *KIAA0753*. Tibial dysplasia is an associated clinical feature of OFD syndrome type IV that is caused by variants in *TCTN3*^5^. Case 5 had both tibial dysplasia and molar tooth signs, revealing an overlap between OFD syndrome type IV and OFD syndrome type VI. We think that OFD syndrome type XVI is difficult to classify because the clinical manifestation is extremely diverse, and the number of cases is not yet sufficient for classification. For the time being, it seems reasonable to consider it as a spectrum caused by the *TMEM107* gene.

Mouse embryos with complete *TMEM107* knockout manifested an embryonic lethal type, exhibiting a broad spectrum of craniofacial defects^13,14^. In our case, the relationship between *TMEM107* and anencephaly could not be investigated because the biological samples of the affected fetuses with anencephaly were not preserved. To the best of our knowledge, there have been no reports of anencephaly in OFD syndromes. These repeated cases of affected fetuses with anencephaly is a very rare pattern, and great care should be taken in interpreting this phenomenon.

## HGV database

The relevant data from this Data Report are hosted at the Human Genome Variation Database at 10.6084/m9.figshare.hgv.3137.
